# Correction: MSPM: A modularized and scalable multi-agent reinforcement learning-based system for financial portfolio management

**DOI:** 10.1371/journal.pone.0265924

**Published:** 2022-03-17

**Authors:** Zhenhan Huang, Fumihide Tanaka

There are errors in the Funding statement. The correct Funding statement is as follows: This work was supported by JST SPRING, Grant Number JPMJSP2124.
