# Supplementary material for: MSPM: A modularized and scalable multi-agent reinforcement learning-based system for financial portfolio management
Source: PLoS One. 2022 Feb 18;17(2):e0263689. doi: 10.1371/journal.pone.0263689 (PMC8856562; doi:10.1371/journal.pone.0263689)
Supplement: S1 Appendix — (PDF) [file pone.0263689.s001.pdf]

S1 Appendix. Model selection for EAM and hyperparameter tuning. For model selection, we have tested different architectures of neural network models for the DQN agent in EAM. Among them, we chose Residual Network with 1-D convolution since it performed the best on the validation dataset described in Table 2 in Data ranges section. We also performed numerous experiments for hyperparameter tuning on the validation dataset to make sure the hyperparameters implemented and stated in the article are the optimized for the use cases in this research.
