# Supplementary figures and images for: MSPM: A modularized and scalable multi-agent reinforcement learning-based system for financial portfolio management
Source: PLoS One. 2022 Feb 18;17(2):e0263689. doi: 10.1371/journal.pone.0263689 (PMC8856562; doi:10.1371/journal.pone.0263689)

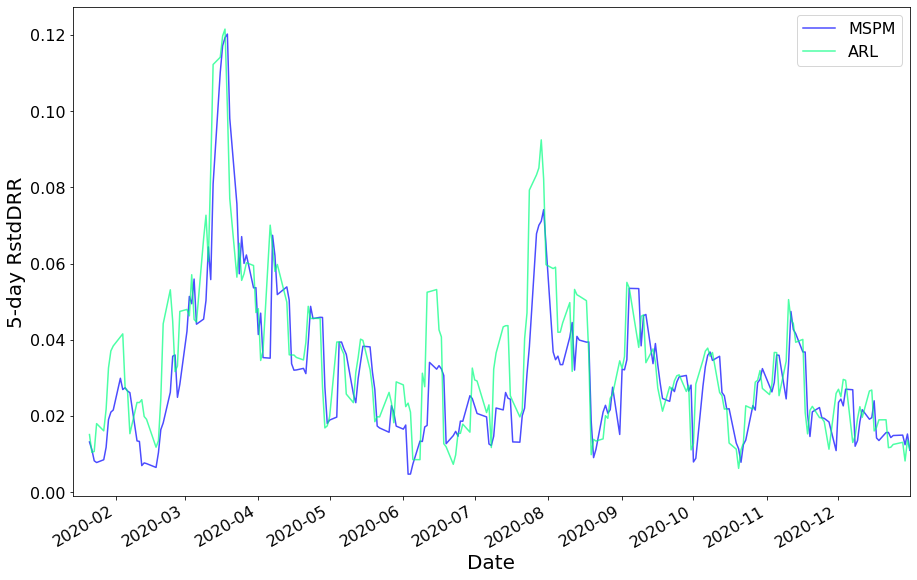

Supplement: S1 Fig — (TIF) [file pone.0263689.s002.tif]

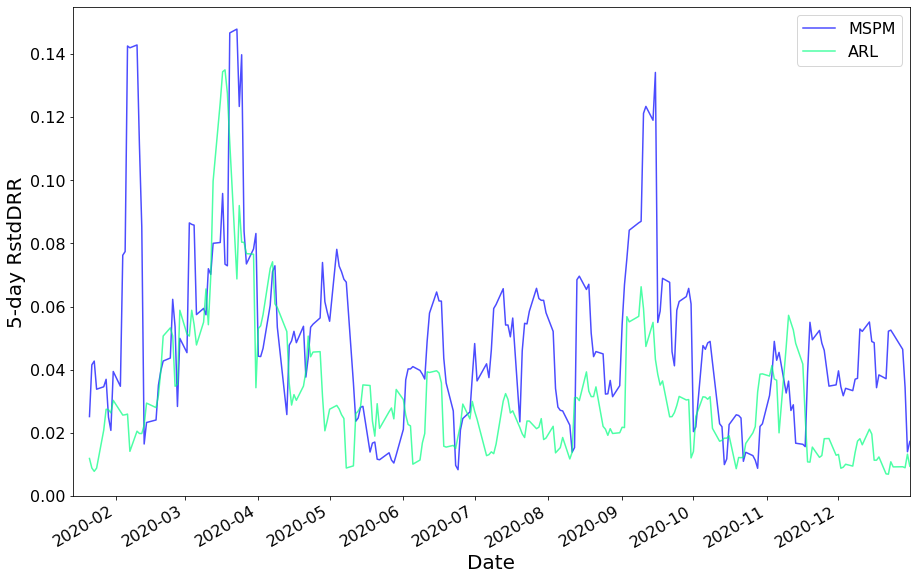

Supplement: S2 Fig — (TIF) [file pone.0263689.s003.tif]

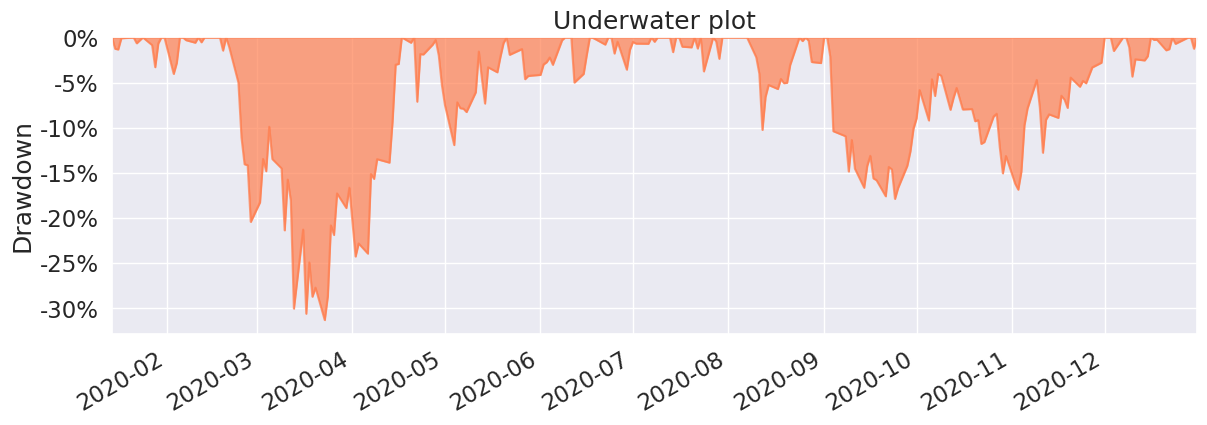

Supplement: S3 Fig — (TIF) [file pone.0263689.s004.tif]

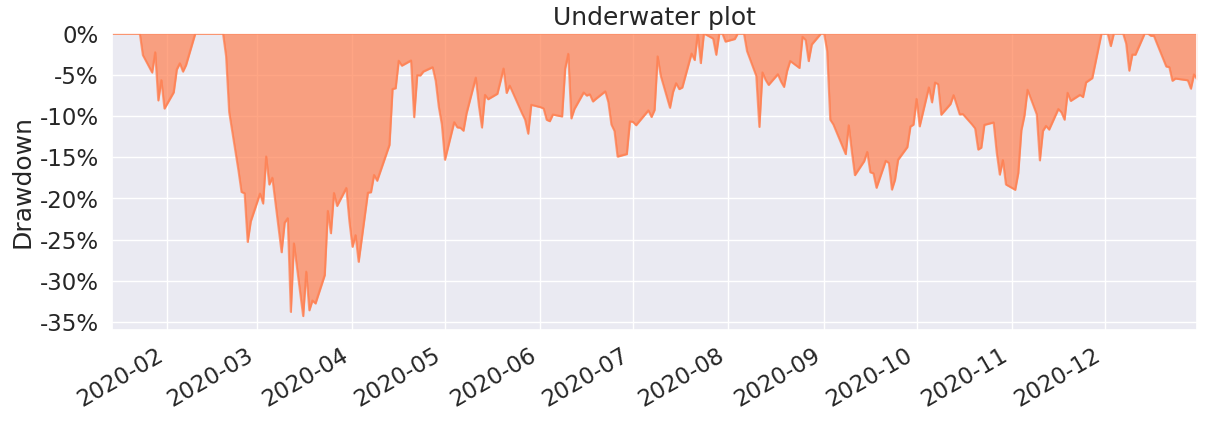

Supplement: S4 Fig — (TIF) [file pone.0263689.s005.tif]

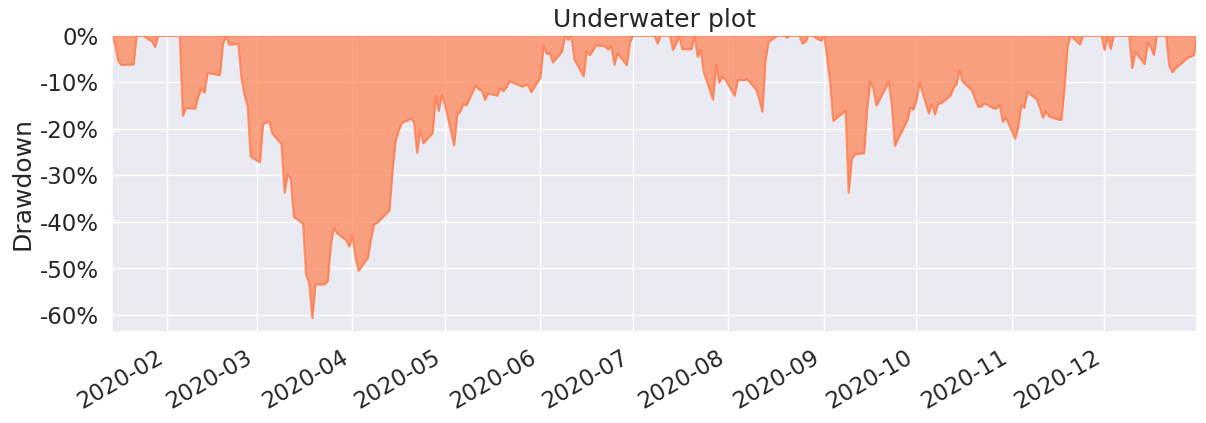

Supplement: S5 Fig — (TIF) [file pone.0263689.s006.tif]

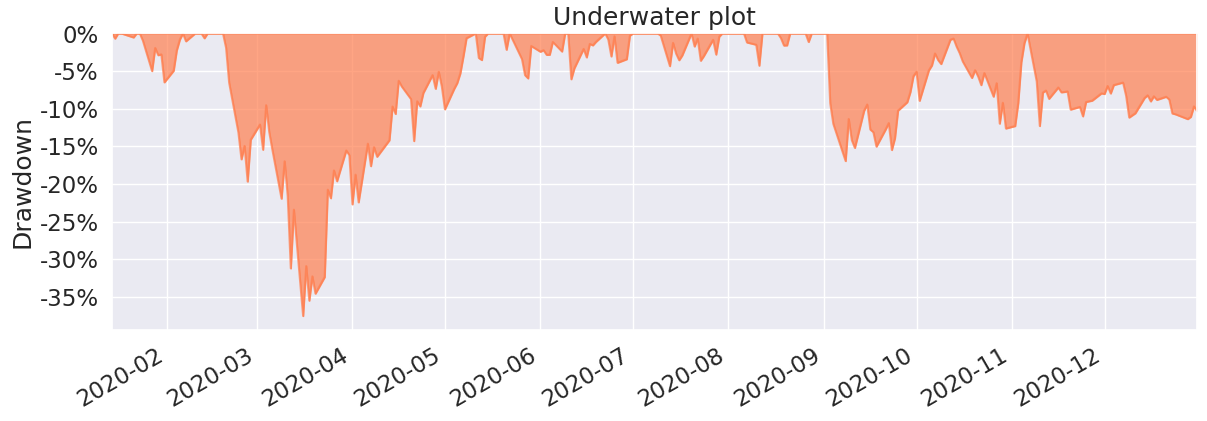

Supplement: S6 Fig — (TIF) [file pone.0263689.s007.tif]
